# Genesis—progression of early jaw osteoradionecrosis in head and neck cancer survivors: dynamics of independent but interconnected factors

**DOI:** 10.3389/fonc.2026.1941783

**Published:** 2026-10-09

**Authors:** Sylvie E. Delanian

**Affiliations:** Département d’Oncologie-Radiothérapie, Assistance Publique des Hôpitaux de Paris (APHP), Hôpital Saint-Louis, Paris, France

**Keywords:** dental extraction, head neck cancer, jaw necrosis, oral microbiota, osteoradionecrosis (ORN), radiation induced fibrosis (RIF), vascular injury after radiation therapy

## Abstract

**Purpose:**

Long-term head and neck cancer survivors are few, but always exposed to a risk of mandibular osteoradionecrosis (ORN) despite the use of appropriate dose-volume constraints in today’s radiotherapy. Here, ORN development is approached from another look, in an attempt to explain its complexity.

**Definitions:**

Early ORN is best defined by length of the mucosal defect (<2 teeth long) and radiological assessment of the depth of areas of bone resorption (lysis below the level of the inferior alveolar canal) in the radiotherapy volume. Technical improvements have greatly reduced the incidence of ORN, but a lifelong risk remains and requires long-term follow-up.

**Causal factors:**

Pathogenic hypotheses have evolved over time from the “radiation-trauma-infection” triad to the 3H theory of “hypoxia-hypocellularity-hypovascularity”, and then radiation-induced fibro-atrophy of the bone. ORN is a dynamic process combining interacting causal factors that worsen each other over time: (a) radiation-induced fibro-atrophy of the bone and its related radiotherapy technical parameters; (b) periodontal disease/dental trauma and their related questions on optimal timing of pre- or post-radiotherapy extractions and how many teeth; (c) infection/oral microbiota and their related mechanically inaccessible bone sequestra (residual bacteria); (d) vascular factors from capillary network destruction to artery stenosis of irradiated branches and the carotid trunk.

**Conclusion:**

Symptomatic medical care focusing on comorbidity parameters and devitalized tissue removal is not sufficient to protect patients against ORN. These new insights should help to treat early ORN through concomitant control of these causal factors and best dental care.

## 1 Introduction

Thanks to progress in oncology, half of treated cancer patients are long-term survivors beyond 10 years of follow-up (1). Radiotherapy (RT), combined with surgery and systemic treatments, is pivotal to achieving cure of head and neck cancer (HNC). However, although modern imaging allows exact adaptation to target tumor volumes and today’s RT uses modulated arc therapy, RT of the oral cavity remains a challenge because of possible long-term severe sequelae, of which osteoradionecrosis (ORN) is the most daunting.

The first clinical observations of ORN of the jaw, which is localized radiation-induced (RI) bone necrosis, were made at the beginning of the 20th century, in the hand (Hawks) and jaw (Freud) (2). The clinical definition of ORN has varied over time based on its evolution, in particular under treatment, without clear formalized consensus until 2025. In fact, ORN’s fearsome reputation resides in its ineluctable progression, when early ORN becomes uncontrolled despite routine care.

What is today’s view of this long-known complication of radiation therapy? Various theories described in the 20th century that implicated radiotherapy techniques, vascular or dental parameters, are often unable, individually, to explain the entire complexity of the disease and therefore its best management. A better insight into the process of ORN should take into account the time factor, which combines waves of successive and/or concomitant attacks by the five causal agent families considered individually in this article.

It is relevant to consider that ORN happens in irradiated fibro-atrophic tissue, in which radiation is, by definition, always causal and present, because the tissue has been irradiated and “never forgets” (3). Then, ORN becomes a local injury because a combination of intertwined causal agent families attacks the “awakened” latent fibrotic atrophy in successive steps until there is no way for the bone to heal. The attacks with this “temporal effect” then result in an unbalanced scar in a vicious circle: different vascular, dental, mechanical, or infectious imbalances destabilize irradiated bone tissue, and their dynamic entanglement leads to mutual worsening (**Figure 1**).

**Figure 1:**
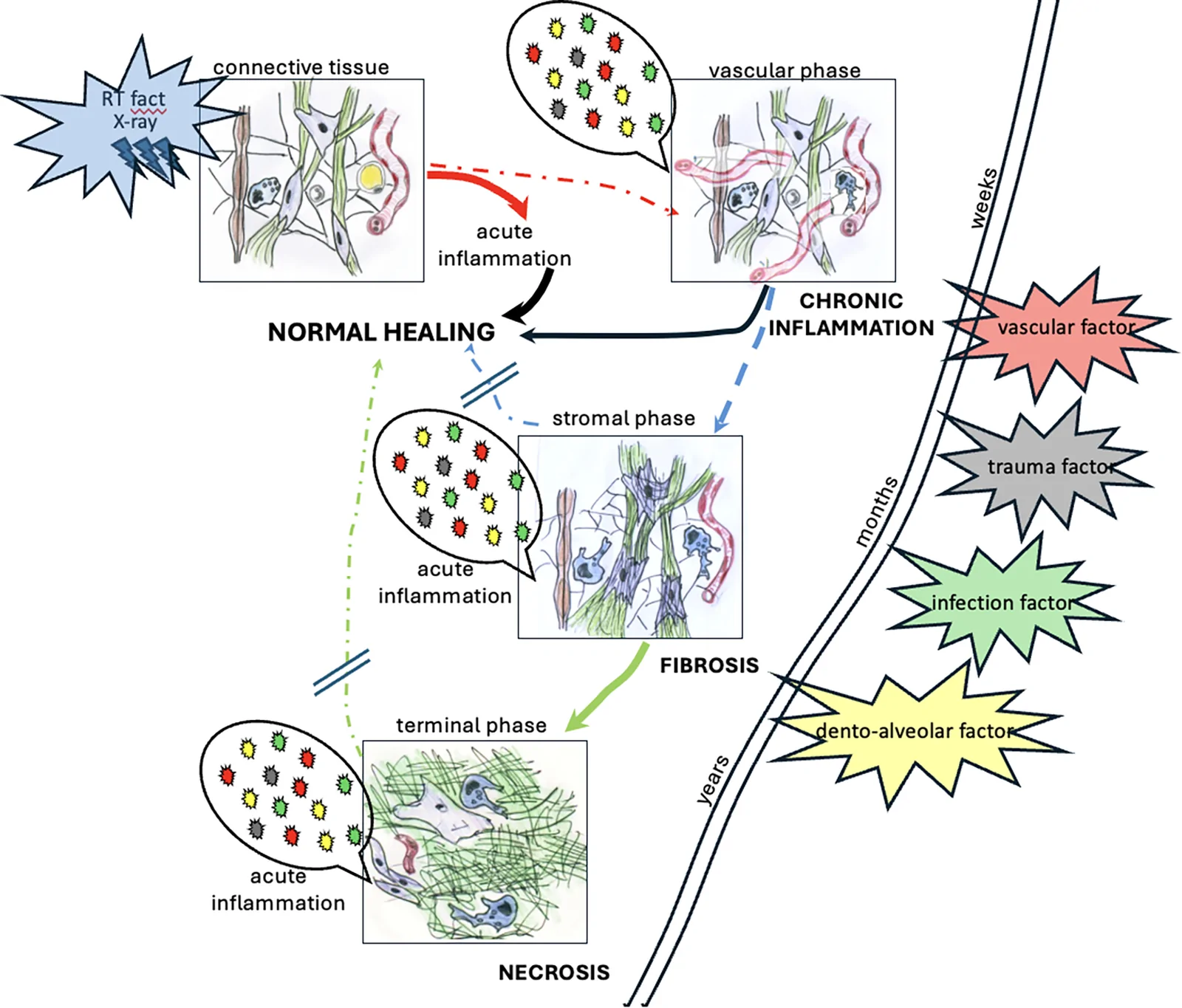
Main pathophysiological stages leading to osteoradionecrosis: concomitant and/or successive attacks over time by causal agent families on “sleeping” irradiated tissue.

It is not known how best to control these causal agent families, usually named aggravating factors, which lead to progression from irradiated bone to early then severe ORN. We examined the existing literature and presented a multidisciplinary and comprehensive review of ORN with a focus on early ORN stages unbalanced by pivotal factors.

Control of these co-agents, if any, with the concomitant use of an antifibrotic treatment should resolve most cases of “early ORN” by allowing reversal of radiation-induced fibro-atrophy (4)?

## 2 Early ORN definition—classifications

We concentrated on the beginning of mandibular ORN (low grade), when localized, by describing “early ORN” extracted from the main published grading systems in **Table 1** (5–11), which excludes “advanced ORN” cases overwhelmed by pathologic fracture, osteomyelitis, orocutaneous fistula, or irritation of the inferior dental nerve, which require complex and mutilating care.

**Table 1:** ORN staging systems: early stages of ORN described differently over time by authors selecting some aspects to establish a pragmatic scale (exposure to mycosis, radiological aspects, time to healing, treatment used) to improve treatment of patients.

Authors (year)	Stages early ORN/total levels	Patients Early ORN/total pts	Author description of early stage osteoradionecrosis (ORN)
Marx (1983) (5)	Stage I/among three (I-II-III)	9 /58	30 dives of hyperbaric oxygen (HBO) used to attain **mucosa recovery *(response to HBO therapy based on hypovascular-hypoxic ORN)***
Epstein (1987) (6)	Stage I /among three (I-II-III)	4 /26	Resolved/healed with or without pathological **fracture**
Epstein (1987) (6)	Stage II /among three (I-II-III)	11 /26	**Chronic persistent** ***(ORN progression)***
Store-Boysen (2000) (7)	Stage 0-I /among four (0-I-II-III-IV)	3-17 /73	0—mucosal defects only I—radiological evidence of necrotic bone with intact mucosa
Store-Boysen (2000) (7)	Stage II /among four (I-II-III-IV)	37 /73	Positive **radiographic findings with denuded bone** intra-orally **(*Combination of clinical and radiological parameters)***
Notani (2003) (8)	Stage I /among four (I-II-III)	2 /87	ORN confined to **alveolar bone**
Notani (2003) (8)	Stage II/among three (I-II-III)	34 /87	ORN limited to the **alveolar bone and/or above** the level of the inferior alveolar canal (***radiological parameters)***
Delanian (2011) (9)	Stage II /among three (I-II-III)	18 /54	Chronic persistent bone exposure without healing; low **SOMA** (subjective objective management analytic evaluation of injury) score level ***(clinical and radiological continuous scoring system)***
Tsai (2013) (10)	Stage I /among four (I-II-III-IV)	10 /30	Minimal **bone exposure** with conservative management only
Lyons (2014) (11)	Stage I /among four (I-II-III-IV)	28 /85	**<2.5 cm length** of bone affected, asymptomatic **(*clinical-radiological parameters and ORN progression)***
***Total***	***Early ORN/among several levels***	***173 early ORN/413 total cases (42%)***	
Watson (2024) (12)	Stage 0-1 /among four (0-1-2-3)	56-120 (**176**) /**219**	***Clinical and radiological features*** (**ClinRad**)

Bolded values means criteria of measure in early stages.

The main staging systems, developed between 1980 and 2014, describe the ORN population (**Table 1**) depending on the authors’ chosen symptoms and practice (5–11): clinical, as mucosal defect and length of bone affected; radiological, as small superficial alveolar bone, larger deeper areas of bone resorption above the level of the inferior alveolar canal; and/or progression of ORN under conservative management and hyperbaric oxygen therapy.

The term “early ORN” describes simple chronic oral mucosal erosion with bone exposure, within the radiation field, with no healing after more than 3 months, without complication or tumor recurrence (**Figure 2**), and its related radiological signs. A recent review of the literature established by a multidisciplinary international expert panel supported the ClinRad classification system for the characterization of jaw ORN within 15 existing systems from 80 publications between 2004 and 2023 (12, 13), which also define jaw ORN in terms of its clinical and radiological aspects.

**Figure 2:**
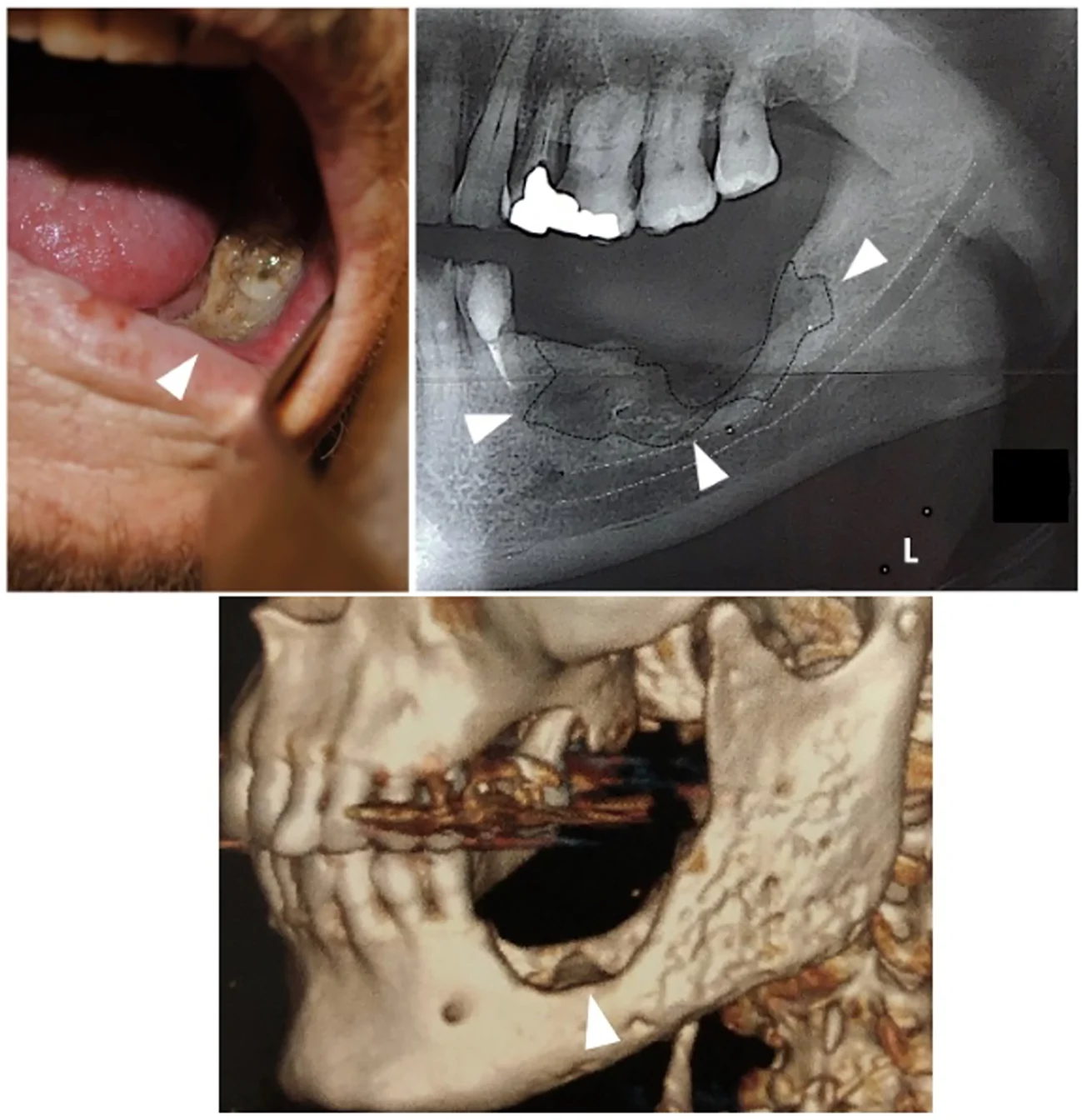
“Early ORN”: endobuccal view, X-ray panoramic, and 3D CT-scan reconstruction.

The clinical symptoms of “early ORN” are well known, albeit described differently by various authors (**Table 1**): ulceration of the lingual mucosa, exposure of the underlying necrotic jaw bone, accompanied by pain and possibly local infection (fetor oris), and related early radiological signs on panoramic radiography (2D) and jaw CT scan (3D) as mandibular extraction sockets, cortical bone interruption, and pagetoid mixed lytic-sclerotic bone changes with/without intraosseous sequestra (**Figure 3**).

**Figure 3:**
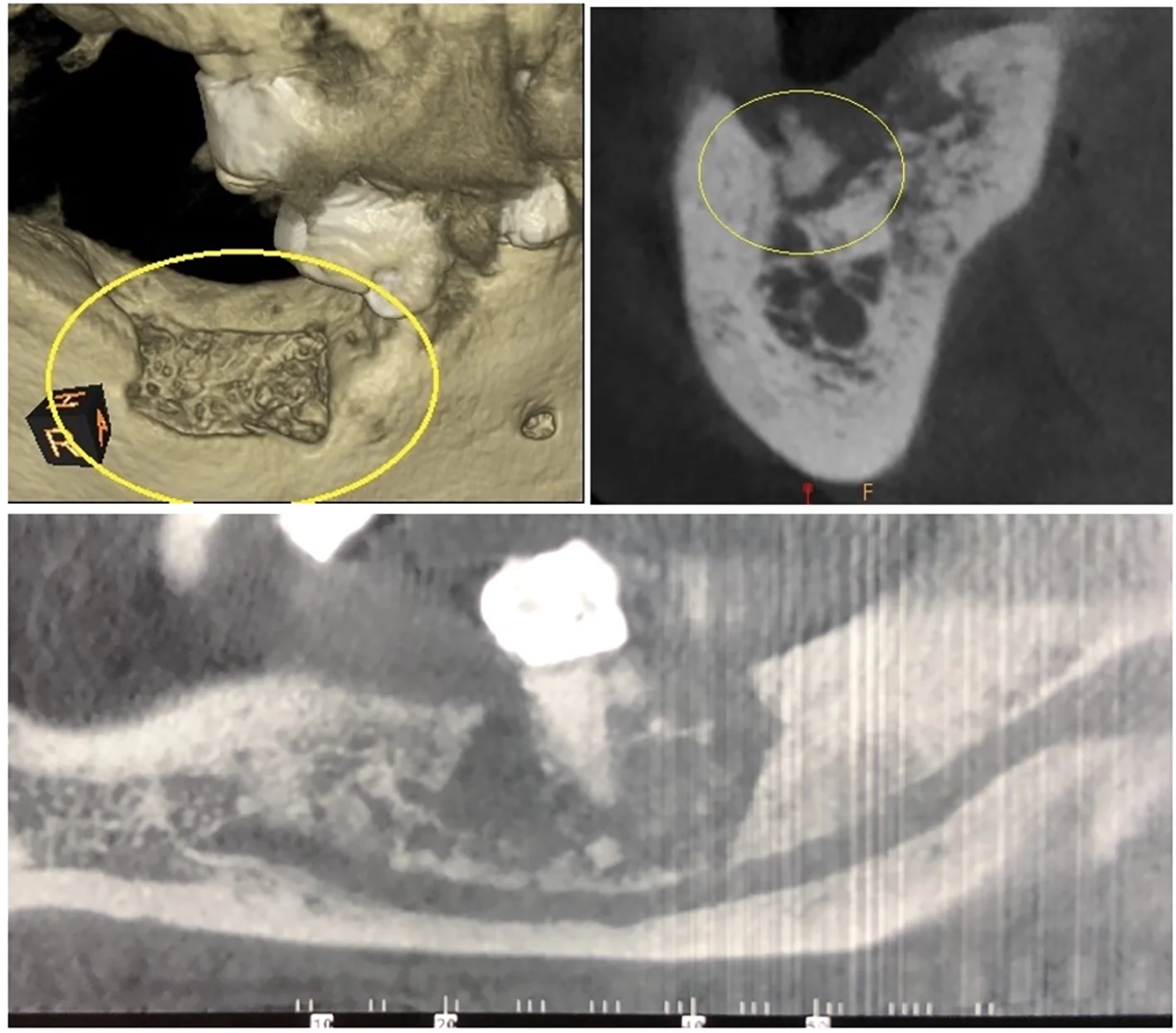
CT-scan radiological signs of “early ORN”: bone sequestrum, bone resorption above tooth, and lysis less than at inferior alveolar canal.

Radiological practice in oncology should expedite diagnosis of early ORN: dental panoramic radiography before RT and every year during follow-up; cone beam CT-scan and arterial Doppler of the supra-aortic trunk every 5 years to detect early ORN and/or correct predisposing factors (see below). Today, jaw CT scan or cone beam CT scan establishes the diagnosis and severity of ORN (14, 15). In a recent retrospective study, positron emission tomography (PET-CT) was used to diagnose advanced ORN with chronic pus and fracture because of inflammation in surrounding soft tissue and to differentiate it from tumor recurrence (16). PET-CT was also able to detect foci in the jaw thereby helping diagnosis of early ORN and its treatment monitoring (17, 18).

These staging systems described the ORN population in the literature, where on average 40% of published cases were early (**Table 1**) classified as “stage I sometimes II,” out of five stages. The early ORN cases extracted from the published studies concerned 2 to 37 cases by study (total of 173 early published cases) among the entire ORN population (early and advanced) of 22 to 87 total cases by study (total of 413 published cases) (**Table 1**).

Today, the most pragmatic ORN classification seems to be described by the ClinRad system, separating “early ORN” and advanced ORN independently of the treatment used (12). and compatible with our experience over two decades helping to treat several hundred patients. It combines clinical length of the mucosal defect (less or more than two teeth long) and radiological assessment of the depth of areas of bone resorption (lysis below or above the level of the inferior alveolar canal, vertical extent of necrosis) (**Figure 3**).

An expert-based consensus by Delphi study clarified recently the need for a standard definition and severity assessment (19). “Although valuable to report and reflect the duration of nonhealing changes observed in irradiated bone, the time or duration of exposed bone is not a mandatory diagnostic feature for ORN” (19).

## 3 Early ORN incidence—epidemiology

Until now, ORN patients treated for HNC were mostly alcohol–tobacco users, of average age older than 55 years, in a male–female ratio of 3:1 (20). Progressive advances in RT technologies and dental care protocols have reduced the estimated risk of ORN in HNC, from 25%-40% in the 1960s to 15%-20% in the 1980s, 10% in 2000, and a mean 5% in 2020 [**Table 2** (10, 21–46)].

**Table 2:** ORN incidence from major retrospective studies with 2–5 years of follow-up, using RT techniques (from EBRT, 3D-CRT, IMRT, VMAT, proton), over seven decades, to treat HNC patients.

Author (institution)	Years of treatment	Site	Radiotherapy technique	Number of irradiated patients	Number of ORN (rate %)	Months RT-ORN interval (range)
Grant & Fletcher; MDA (21)	1954_62	HNC	EBRT	176	66 (**37.5**)	
Murray; MDA (22)	*1966_69 **1971_75	OPC_OCC	EBRT-implant	*249 **404	*61 (24,5) **77 (19.1)	*9 (0-24) **13 (0-48)
Morrish (23)	1971_77	HNC	EBRT	100 *edentulous 22 **teeth 78	22 (**22**.0) *3 (14) **19 (24)	*10 (1-18) **22 (6-72)
Withers (24)	1976_85	OPC	EBRT	676	32 (4.7) *[5.5%/4y;9.9%/8y; 12.5%/10y]*	52 (1-106)
Reuther (25)	1969_99	HNC	EBRT & 3D-CRT	830	68 (**8.2**)	9-14
Chang (26)	1987_2002	OPC	3D-CRT	413 *tooth extraction 163 **edentulous 128 ***dentulous 122	37 (8.9) *25 (15) **1 (0,8) ***11 (9)	9 (1-168)
Lee (27)	1990_2000	OPC_OCC	3D-CRT	198 *109 OPC **89 OCC	13 (6.6)	22
Huang (28)	2000_04	OPC	IMRT	71	1 (1.4)	14
Gomez (29)	2000_07	HNC (46% OPC_OCC)	IMRT	168 *41 OPC **36 OCC	2 (1.2)	31; 32
Gevorgyan (30)	2003_09	OPC_OCC	3D-CRT & IMRT	1575	14 (0.9)	26.8
Tsai (10)	2000_08	OPC	3D-CRT (17%) IMRT (83%)	402	30 (7.5)	8 (0-71)
***Nabil*** (31 (META-ANALYSIS: *22 randomized trials)*	***1985_05***	***HNC***	***3D-CRT & IMRT***	***5742***	***117 (2)***	
De Felice (32)	2009_14	HNC	3D-CRT (11%) IMRT (89%)	653	36 (5.5)	6 (1-53)
Kuhnt (33)	2003_13	HNC (63% OPC_OCC)	3D-CRT IMRT	776	51 (6.6) *12 OPC **34 OCC	9 (0-90)
Raguse (34)	2004_09	HNC (91% OPC_OCC)	3D-CRT (70%) IMRT (30%)	540	38 (7)	14,5
De Maesschalck (35)	2000_12	OPC	*3D-CRT (62%) **IMRT (38%)	234 *145 **89	25 *16 (**11**) [4.8%/3y] **9(**10.2**) *[8.9%/3y]*	
Kuo (36)	2000_13	HNC	IMRT	1759 *edentulous 522 **teeth 1237	39 (2.2) *27 (5.1) **12 (0.97)	36 *3.25 ± 2.24 (0.62-8.89 y **2.51 ± 1.6 (0.69-5.84)
Studer (37)	2002_13	OPC_OCC	IMRT	531 *441 OPC **202 OCC	36 (7) *16 OPC (5) **19 OCC (12)	20 (1-104)
Chen (38)	2001_11	OCC	IMRT	1692 *228 OPC **1464 OCC	105 (6.2) *3 (1.3) **102 (6.96)	29.8 ± 22.9
Owosho; MSK (39)	2004_13	OPC-OCC	IMRT	1023 *724 OPC **299 OCC	44 (4.3) *32 OPC **12 OCC	19 (0-120) *14.6 **36,1 (p=0.02)
Caparrotti (40)	2005_14	OPC	IMRT	1196	71 (6.0) *[3%/1y; 5%/3y; 7%/5y]*	11
Moon (41)	2009_15	OPC-OCC	*3D-CRT (11%) **IMRT (89%)	252 *184 **68	14(5.5 = 3.8-7.0) *5 (19) **9 (4)	8 (3-40)
***Mohamed; MDA*** (42) (CASE CONTROL)	***2002_13***	***OPC***	***IMRT***	***199*** ***(1:2)*** *131 controls*	***68***	***16***
***Aarup-Kristensen*** (43) (CASE CONTROL)	***2007_15***	***HNC***	*3D-CRT (6%)* ***IMRT (94%)***	***178*** *on 1224* ***(1:2)*** *122 controls*	***56 (*** *4.6)*	***10.9***
Zhang; MDA (44)	2011_14	HNC	*IMRT **Proton	*534 **50	*41 (7.7) **1 (2)	*11.4 (6.7-16.1)*
Watson; Princess Margaret Toronto (12) *prospective*	2011_17	HNC	IMRT	2732 (*490 OPC-* *969 OCC)*	219 (8%) *(73 OPC-* *109 OCC #12.5%)*	
***Yang; Mayo*** (45) (CASE CONTROL)	***2013_19***	***HNC***	*VMAT **Proton	*931 (905 controls with 503 OPC-OCC) **335 (326 controls *with 153 OPC-OCC)*	*26 (4.91) *[# 26 OPC-OCC]* **9 (4,97) *[# 8 OPC-OCC]*	
Singh; MSK (46)	2013_19	OPC	Proton	122	13 (10.6) *[5.2%/3y; 11.5%/5y]*	*13.5 (0.2-58.0)*

ORN, osteoradionecrosis; HNC, head and neck cancer; OPC, oropharyngeal cancer; OCC, oral cavity cancer; 3D-CRT, conformal radiotherapy; IMRT, intensity-modulated radiotherapy. Bolded values means clinical trial versus control.* and ** patients subgroups and their related results.

A systematic review of 22 randomized controlled trials showed 117 ORN cases among 5,742 HNC patients: the estimated incidence rate of ORN (1985-2010) was increased by 2% with additional risk factors (31).

Differences in published values of ORN incidence are related to study heterogeneity (**Table 2**) (10, 21–46): different follow-up (interval between RT and ORN), HNC subtype (with or without oral cavity), RT techniques (orthovoltage of the 1960s, megavoltage of the 1970s, 3D-CRT conformal radiotherapy of the 1990s, IMRT intensity-modulated radiotherapy after 2000), aims of RT (adjuvant postoperative or non-operated tumor), type of pretreatment dental care, and early ORN not always included in the reports (no universal classification).

In fact, the risk of ORN is lifelong, as for any irradiated tissue: observable, by definition, if patients are long-term survivors. The actuarial incidence of mucosal and bone complications is described as a continuous variable (without peak of incidence). For example, in a 10-year retrospective study of 142 ORN cases, the incidence was 38% at 3 years, 28% at 3–6 years, 15% at 6–9 years, and 18% at >9 years (20). ORN incidence also depends on the primary HNC subsite [**Table 2**; (10, 21–46)]. ORN rates in oropharyngeal cancer (OPC) and oral cavity cancer (OCC) are described as higher than other head and neck cancers. In fact, defective healing and fibrotic risks are enhanced by cumulated events in the oral cavity: site of the tumor in the oral cavity, mechanical trauma of biopsy and surgery, septic dental care, and higher mandibular RT dose and volume (**Table 2**). The introduction of more targeted RT has helped to reduce ORN risk: Tsai et al. reported the incidence of ORN decrease after IMRT, but it was still 6% in the oropharyngeal cancer cohort (10), whilewhereas there was no reduction in ORN rates for oropharyngeal cancer patients when comparing IMRT with 3D-CRT in other studies (35, 42). Caparotti et al. reported ORN rates in the oropharyngeal cancer cohort of 3% at 1 year, 5% at 3 years, and 7% at 5 years (40), an incremental increase which was confirmed by Habib et al. who reported respective values of 4.7%, 9.4%, and 10.5% (47), suggesting that pooling all HNC patients in the same analysis will dilute the message, whilewhereas the nature and severity of ORN depend on various factors (48).

Thus, although the incidence of ORN has markedly decreased every decade thanks to improvements in RT technique and dental care, it seems to have reached a plateau (**Table 2**) (10, 21–46)), as confirmed recently (49).

What is new in terms of causal agents, long known in the ORN literature as the “three Ws” temporal effect, “who, when, how” and “tooth-bone-artery”? A first theory regarding ORN focused on three main factors: RT dose, local injury, and infection (50). This was confirmed in 1970 by the Meyer triad “radiation-trauma-infection,” also called “radiation osteomyelitis” (51). In 1983, because evidence of trauma was lacking in one-third of ORN cases, Marx elaborated the “3H” theory of “hypoxia–hypocellularity–hypovascularity” and concluded that “ORN is not a primary infection of irradiated bone, but a complex metabolic and homeostatic deficiency of tissue that is created by radiation-induced cellular injury; micro-organisms play only a contaminating role in ORN, and trauma may or may not be an initiating factor” (5).

In fact, ORN occurrence involves *five* interlinked causal agent families whose proportions vary from one patient to another (personal contribution (52)) and for each patient during his or her own disease progression: each parameter, as radiotherapy, dentition, trauma, infection, or vascular, has been a causal agent or simple aggravating factor and vice-versa.

## 4 Causal agent 1: bone radiotherapy-related factor

How to think of the impact of radiotherapy parameters in ORN occurrence?

### 4.1 Radiotherapy—technical factors

ORN is, by definition, located in an irradiated volume. Various radiotherapy (RT) parameters are involved in its incidence and severity (10, 39, 42): conventional versus accelerated-hyperfractionated RT; aims of RT with postoperative RT versus concomitant chemotherapy-RT; re-irradiation in a given volume; oral brachytherapy (with risk of hyperdose sleeve against bone structure). In fact, RT risk factors involve technical parameters of mandibular vulnerability because of the heterogeneity of RT “concentration”: a high RT dose in a small volume, inside a large volume at moderate total RT dose, especially for oral cancer.

The radiation delivery mode has over decades modified RT dose–volume parameters in jaw bone for a given target tumor dose–volume, namely, orthovoltage between 1966 and 1972 with a mean 37% ORN occurrence, megavoltage RT (1970-80) with 14%-22%, conformal 3D-CRT (1990) with 5%-8% (**Figure 4**), IMRT since 2000 with 2%-5%, and then VMAT or protons (35, 45, 46, 53). Despite the undeniable advantages of IMRT-VMAT in HNC, “beam path” toxicity can occur in extended non-targeted areas (low doses in the whole mouth including the anterior mandible), unobserved before with opposite lateral beams (54). Proton therapy limits the dose to adjacent structures thanks to focal deposition of the Bragg peak. This greatly lowered (by a mean 40%) the jaw RT dose in HNC, without modifying the maximum RT dose and ORN risk (55).

**Figure 4:**
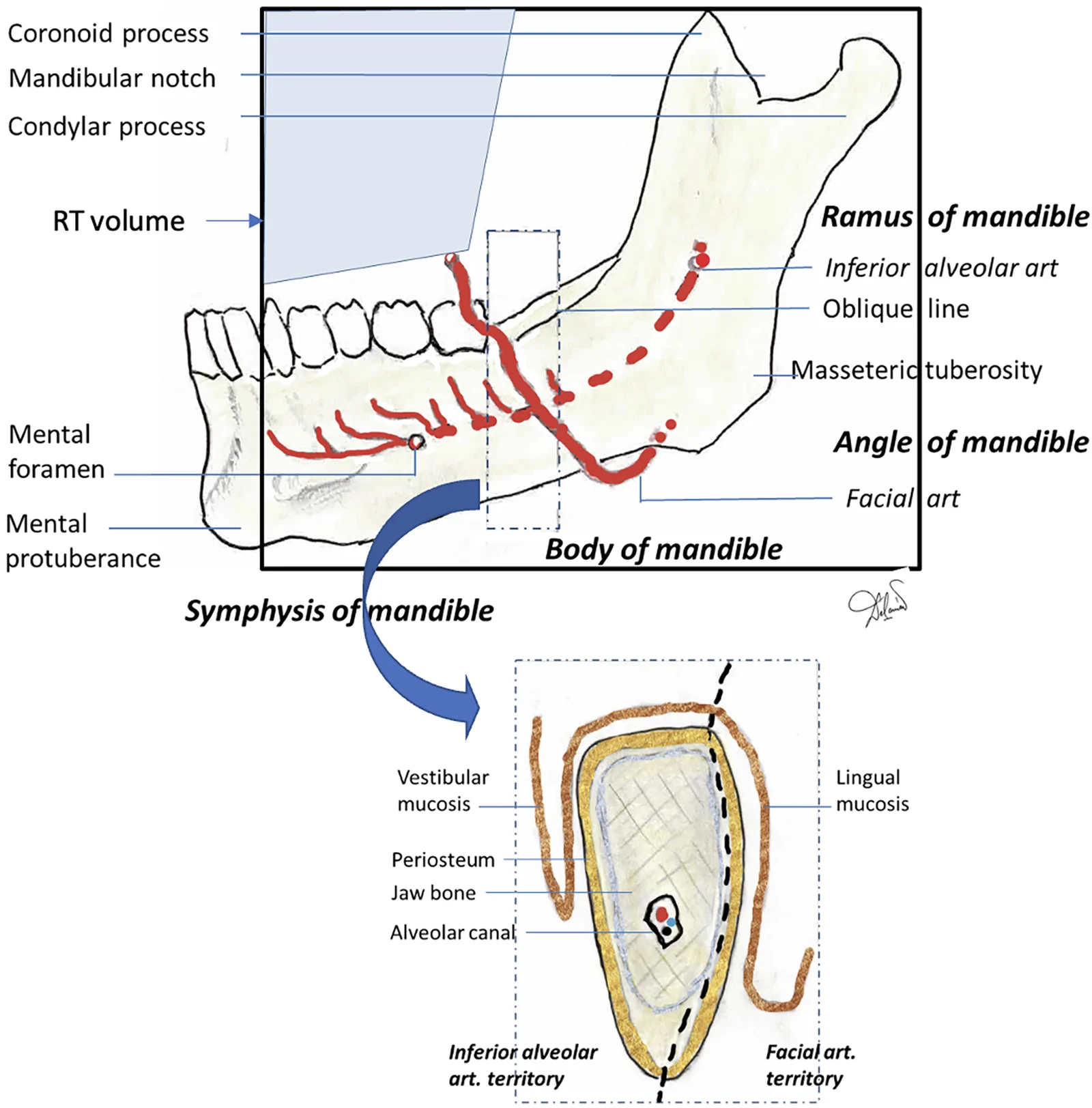
Schematic beam path including jaw bone and carotid artery branches (external view and mandibular cross section) after conformal RT of the oropharynx.

There is no international agreement on a critical RT dose that leads to ORN. Rubin and Casarett described a TD 5/50 (total dose causing 5%-50% complications) of HNC occurrence at 60–90 Gy (56). Bedwinek et al. observed no ORN <60 Gy, 1.8% ORN at 60–70 Gy, and 9% ORN >70 Gy (57). The dose-dependent risk of ORN agreed today is dose <40 Gy with <6% ORN, 40–60 Gy with 14% ORN, and >60 Gy with 20% ORN (58). While dose >60 Gy is the most significant predictor of ORN in most studies (59–61), dose >40 Gy puts patients at risk of ORN (54) irrespective of other agents (62). A small volume of high RT dose in bone is known to be an ORN risk. Dosimetric evaluations in ORN patients were not clearly different to those in non-ORN patients, showing Dmean 49.0 Gy, Dmax 71.7 Gy, and mandible dose–volume histogram V40 69%, V60 45%, and V70 17%. Patients treated by 3D-CRT had significantly higher mean Dmax 74.5 Gy versus 70.2 Gy after IMRT, V60 (65% vs. 33%), and V70 (36% vs. 7%) (41). As oral cancer and the dentition are physically close, the mandibular third molar and second molar may receive on average a dose >60 Gy, which may explain why pre-RT dental extraction cannot eliminate the ORN risk (63).

Besides that, a large volume of low RT dose after IMRT might also contribute to ORN genesis: between ORN patients and matched patients, there was a significant difference in jaw Dmean (48.1 Gy vs. 43.6 Gy), but not in Dmax (74 Gy vs. 73.2 Gy) (38). Teeth receiving 30–60 Gy are more susceptible to physical injury, and microhardness is reduced at the cervical enamel junction, thus contributing to RI caries (64–66). More recently, an RT dose >30 Gy across the whole dentition on the RT dose map (whole mouth) illustrates its vulnerability to dental disease and the subsequent development of ORN because of the absence of unirradiated bone volume (47). Frankart et al. suggested that the volume of lower-dose radiation contributed to ORN more significantly than a maximum point dose (55).

Normal tissue complication probability prediction of ORN based on dose–volume parameters was developed to guide RT mandibular dose constraints: for an ORN risk lower than 5%, <30% of the jaw should receive a dose ≥35Gy (67).

### 4.2 Bone histopathology: a radiation-induced pagetoid bone structure

Normal mature bone is a well-known hard tissue, inert in appearance, mostly made of extracellular matrix including organic collagen, inorganic crystallized calcium phosphate, and some specialized cells. Bone remodeling is determined by mechanical and metabolic locoregional factors, with constant reshuffling by osteoblastic apposition—osteocytic resorption balanced with activated osteocytes (68, 69).

ORN schematically follows three histopathological phases that lead to a fibro-atrophic bone with cellular and matrix destruction compensated by deficient osteogenesis and scar tissue (3, 70, 71). In the forming phase, irradiated bone tissue progressively and non-specifically atrophies and is normally calcified, corresponding to radiological osteoporosis, in which asymptomatic tissue is weakened and always in equilibrium. In the constituted phase (early ORN), because of several imbalances, there is progressive bone fibro-atrophy in a small volume, showing direct damage as hypocellularity, altered bone matrix, and indirect damage as fibrotic compensation and altered vascularization, leading to pagetoid radiological signs (3, 72).

Hypocellularity reflects defective healing: imbalance between proliferative and quiescent bone cells, combining mosaic areas of osteogenesis and extended areas of osteolysis. Dambrain has well described the multiple types of radiation-induced bone tissue destruction (72): (a_1_) peri-osteocytic pathognomonic osteolysis (73), bone resorption with peripheral haloes around osteocytes due to lysis of glycosaminoglycans and then collagen alteration and leakage of mineral salts*;* (a_2_) osteoclastic resorption by macrophagic action followed by late neo-osteogenesis; (a_3_) osteoporosis due to premature aging, osteocytes reaching the end of their lifespan without replacement, amplified by (a_4_) local bacterial destruction; and (a_5_) chemical resorption, involving surface dissolution without cell intervention by modified saliva or purulence.

b- Weakened bone matrix reflects imbalance between its synthesis and degradation, leading to poorly calcified bone.

c- Radiation-induced fibrosis reflects excessive healing with scar tissue compensation (3, 68). The mechanisms consist of a vicious circle built on fibroblast proliferation, matrix deposition, and cytokine amplification. Whereas reactive oxygen species are involved in physiological functions (differentiation, proliferation, and inflammation), their excess production results in pathological stress on tissues as induced by physicochemical damage, infectious agents, and deficient antioxidant defenses and fibrogenesis becomes possible. Subsequent additional stress, chronic or brief and repeated resulting in abnormal radical concentrations, helps to extend and intensify the process.

d- Impaired vascularization. Hyperemia, endarteritis, and vascular thrombosis begin soon after RT, with acute inflammation (74). Hypovascularization and fibrosis occur after 6 months with progressive ischemia of bone, which is rather susceptible to stress. Presumably, the unirradiated segments contribute to the repair of the damaged portions. However, secondary damage may result from ischemia, fibrosis, and denervation (68, 69).

Moreover, after surgery for localized ORN, the histological omnipresence of a necrotic area and/or inflammation-abscess at bone margins may raise questions about the anatomic limits of ORN, corroborated by the frequent persistence of clinical ORN despite radical dissection (75). For example, ORN was controlled in 95% of cases by 8-week antibiotic adjuvant postoperative therapy, versus 60% after ORN surgery alone (76).

In the advanced phase of osteonecrosis, defective healing fistula, fracture, and osteomyelitis are observed (not developed in the present work).

In summary, modern RT has made it possible to optimize the dose–volume technical parameters in the jawbone, considerably reducing the risk of ORN, without being able to eliminate it. Because of the oncological need for mouth irradiation, each millimeter of PTV must take into account the mechanical barrier properties of the mandible limiting the risk of tumor spread, and the risk of a high curative dose, even if small volume, or a large volume at a prophylactic dose. ORN shows fibro-atrophic tissue with cellular destruction poorly compensated by deficient osteogenesis and hypovascularization.

## 5 Causal agent 2: dentoalveolar-related factor

How should we assess the impact of dental parameters on the onset and worsening of ORN today? Before and after radiotherapy, the identification of dentoalveolar bone disease reflects both septic and traumatic factors affecting teeth (periapical disease) and/or gums (periodontal disease), possibly allowing for the occurrence of ORN (77).

### 5.1 Pre-RT dental status of HNC patients

Pre-RT dental caries is a significant known factor in ORN (p=0.047) (78). Dental decay is found in 31% of the general population of England compared with 71% (79) and 61% (80) in HNC patients. Pre-RT periapical disease is considered as an independent ORN risk factor (HR 6.3 (3-13.2) p<.001) which increases 3.5-fold the 5-year cumulative ORN incidence (81). Periapical disease is prevalent in all HNC studies, ranging from 40% (78) to 80% of patients (80, 82). Bone loss is reported in 66% of HNC patients versus 45% in the general population (79).

Thus, before RT, a careful dental examination (clinical and panoramic radiography) should be carried out to assess teeth (third molar), carious/restored teeth, and horizontal bone loss, which identify any preexisting disease: RCSE guidelines 2026 recommend extraction of any teeth directly involved with the tumor or with a poor long-term prognosis (unrestorable caries, deep periodontal pockets, and non-vital teeth) to limit ORN occurrence (cf infra VI.1).

### 5.2 Radiation-induced caries and periodontal disease

After RT, radiation-induced damage to dentition (**Figure 5**) progressively concerns periapical (crown–root caries) and periodontal disease (gum and alveolar–tooth ligament). Thus, it precedes occurrence of early ORN by direct and/or indirect RI oral imbalance. [reviewed in 83 & 84].

**Figure 5:**
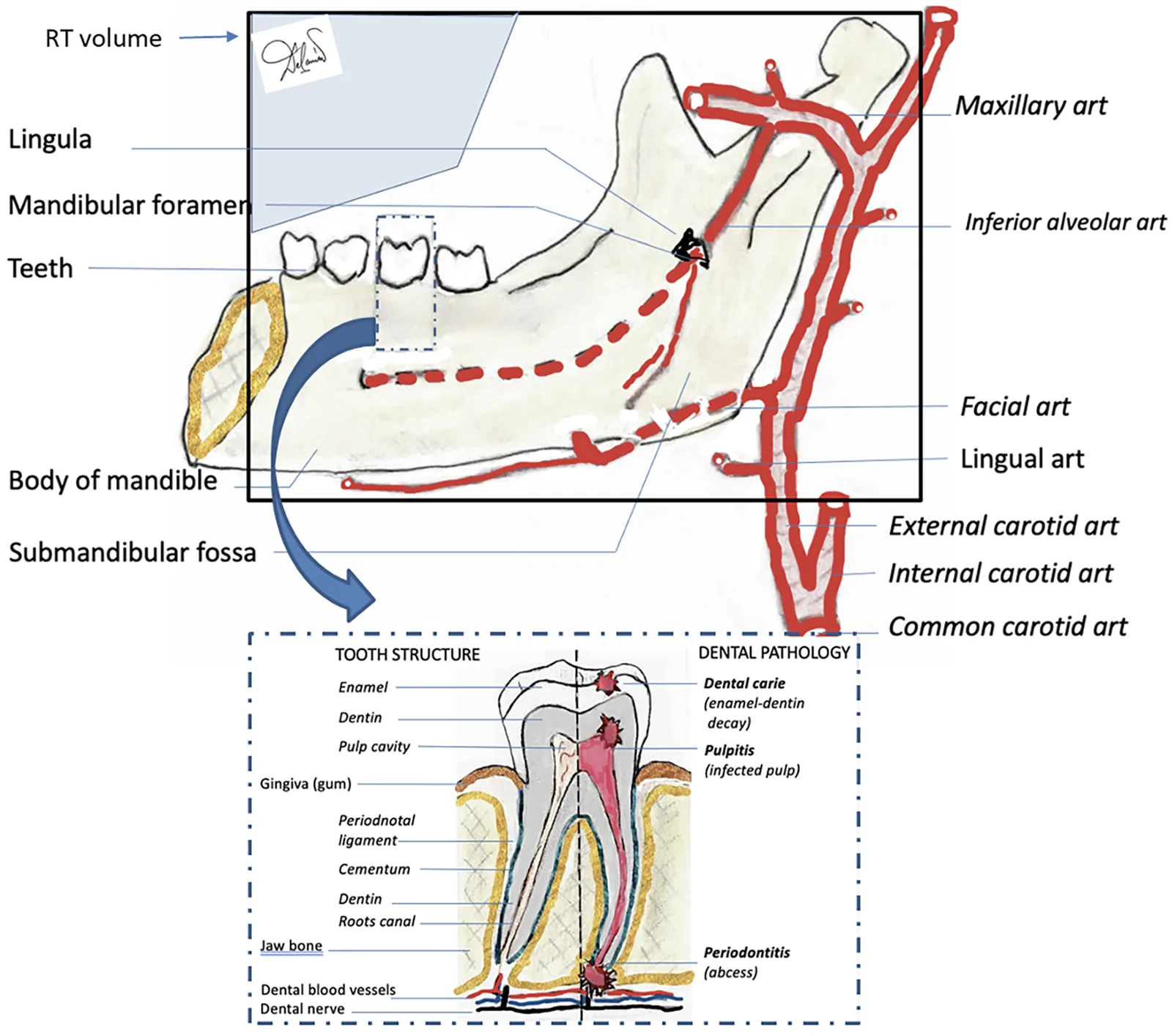
Schematic beam path including jaw bone and tooth structure (internal view and dental cross section) after conformal RT of the oropharynx.

A recent meta-analysis of 15 studies (publications 1975-2019) showed that the incidence of dental caries was 29% after RT and increased significantly after higher RT doses (respectively, 15% for 60 Gy; 26% for 65 Gy; 46% for 70 Gy) or combined chemotherapy (83). The importance of periodontal disease seems currently understated, as it increases the risk of ORN by 19%-33% (85, 86).

Radiation-induced early dental lesions progress from the tooth enamel (caries) to the dentin and then to the pulp cavity, whereas RI periodontitis progresses from the gums to the alveolar–tooth ligaments and then to the alveolar bone (**Figure 5**). The National Cancer Institute-CTCAE classification specifies three grades of dental caries (periapical disease): caries not involving the root (Grade 1), caries involving the root (Grade 2), and caries resulting in pulpitis or periapical abscess or resulting in tooth loss (Grade 3). The same classification also specifies three grades of periodontal disease: Gingival recession or gingivitis; limited bleeding on probing; mild local bone loss (Grade 1); moderate gingival recession or gingivitis; multiple sites of bleeding on probing; moderate bone loss (Grade 2); spontaneous bleeding; severe bone loss with or without tooth loss; osteonecrosis of maxilla or mandible (Grade 3).

Severity of RI damage to dentition depends on direct irradiation of teeth when in the path of the radiation beam and on indirect irradiation of the salivary gland (via hyposalivation).

In direct irradiation of teeth, there are no specific histological changes. The microhardness of dentin is affected: disruption, then a gap, in the enamel–dentin junction resulting in the breaking off enamel chips, followed by microbial colonization.

An indirect but severe effect on RI dentition injury concerns hyposialia, a gradual marked (90%) fall in salivary flow rate seen from the onset of RT. This hyposalivation is partly reversible depending on the RT dose–volume, and whether salivary glands are included. This quantitative saliva parameter is better controlled today. Salivary composition is also modified after RT completion and promotes the onset of caries: increased viscosity, pH reduced to 5, altered electrolyte concentrations which lead to dissolution of the minerals of the enamel and then dentin. In parallel, the shift of oral microbiota to cariogenic bacteria (*Streptococcus mutans*, *Lactobacillus*) and Candida species results in a large increase in periodontal infection. With hyposialia, RI caries starts on the labial surface of the mandibular anterior teeth (no longer cleaned mechanically by the flow of saliva), and then progression affects the cervical area of the tooth, followed by black color changes, and then increased tooth friability until complete amputation of the crown.

Restorative management of radiation caries is challenging due to compromised bonding secondary to radiation-induced alterations in tooth structure (cf infra VI.2).

Thus, RI dentoalveolar deterioration (caries and periodontal disease) is progressive from the tooth–gum interface to the jawbone (direct impact of the radiation beam), which is weakened by bone demineralization, and the deterioration is worsened by indirect hyposialia and its infectious consequences.

## 6 Causal agent 3: trauma-related factors

How to think of the impact of traumatic parameters on the occurrence of ORN?

Trauma, such as dental extraction, and local trauma (dental braces) or any surgical procedure is partly implicated in the genesis and/or development of mandibular ORN, even if it occurs spontaneously (87, 88). The timing of dental extractions in patients undergoing RT remains a pivotal question that is still debated in reviews of many heterogeneous studies (89). There is agreement that dental caries, like its care, present a risk. Only 11% of HNC patients do not require any pre-RT dental care (90). There is disagreement concerning dental extractions to minimize ORN risk: how many? pre- or post-RT? Do edentulous patients have a lower incidence of ORN than dentate patients? (91, 92).

### 6.1 Pre-RT dental extractions

To reduce the risk of ORN, it is accepted that non-functional teeth or teeth with a poor restorative prognosis inside the RT volume should be extracted, with a mean 21 days of healing prior to commencing RT (minimum of 10 days accepted). Dentate patients needed <5 extractions in a mean 60% of cases and >5 in the remaining 40%, for molar teeth in two-thirds of cases (80).

In a univariate Cox model analysis, pre-RT (HR 1.12; 95%CI 1.02-1.24, p=0.01) and post-RT extractions (HR 0.98; 95%CI 0.96-0.99, p <0.007) were associated with a significantly higher ORN risk, whilewhereas the use of steroids (HR 0.68; 95%CI 0.54-0.86, p=0.001) and pre-endodontic treatments (HR 1.12; 95% CI 1.02-1.24, p=0.01) was associated with a significantly lower risk, based on a cohort of 23,732 HNC patients, 1,719 of whom underwent irradiation between 1995 and 2011 (89). It was suggested that the need for extractions post-RT is reduced by thorough dental care before RT. After adjustment for confounders, pre-RT extraction turned out to be insignificant. This was confirmed in a recent meta-analysis of pre-RT extractions including 14,389 extractions in 36,294 patients (40%) in 21 published studies (1983-2019), with an ORN incidence of 5.5% (95%CI, 2.1 to 10.1%) (89).

Prevention of ORN by pre-RT dental extractions of restorable or healthy teeth is not supported by the literature (91), and the risk might increase, in particular for healthy teeth extractions (93). Pre-RT dental extraction, in 413 irradiated patients between 1987 and 2004, did not reduce the risk of ORN for teeth inside the RT volume: ORN rate was <1% if edentulous; 15% if pre-RT extraction; 9% without extraction (26).

Patel et al. recently described, in HNC before RT, a reduced number of teeth and of third molars as the other main ORN factors. Active smokers have significantly fewer teeth (p=0.01), including third molars (p=0.007), and greater horizontal bone loss (p=0.0000) than non- or ex-smokers (85). Furthermore, the oropharyngeal cancer profile has been modified by major growth in human papilloma virus infection, which has been epidemic since 2008, along with a decline in the smoking-alcohol rate and better dentition: mean 25.6 teeth/OPC in the general population of England versus 19.8 teeth/HNC, and a higher number of carious/restored teeth (47). Most studies are historic and do not reflect the changing pattern in tooth retention: in the 1990s, in 947 HNC patients, 59% were edentulous, 14% had poor dentition, and 18% had intact dentition (94) compared with a 2013 cohort with only 2% of edentulous HNC patients (80).

Pre-RT dental extractions (>8 teeth) that reduce the number of teeth below the threshold of functional dentition (less than 21) (80), coupled with the highest RT dose level in OPC, expose the unhealed extraction sites to higher ORN risk (26, 95, 96). There is a trend to increased incidence of ORN when more than four teeth are removed, especially in current smokers (85).

In summary, non-functional or poor restorative teeth inside the RT volume should be extracted, and not restorable or healthy ones, related to a local traumatic risk greater than the infectious risk. Pre-RT dental extractions (>8; <21) with a high RT dose level in OPC exposes the patient to the risk of unhealed extraction sites, especially in young human papilloma virus-positive patients with improved survival.

### 6.2 Post-RT dental extractions

Post-RT dental extractions reviews in 81, 83, 88, 97, 98 can be divided into extractions performed soon after RT or at 6 months after the start of radiotherapy. Analysis of 93 ORN cases treated between 1972 and 1992 showed that 89% were trauma-induced and 85% had mandibular ORN in the body and angle regions (74).

ORN occurrence has for decades been described as trauma-induced after dental extraction in two-thirds of cases. In a study of 536 ORN cases, Marx described 61% as trauma-induced versus 39% as spontaneous. 84% (274/327) of trauma-induced ORN cases related to dental extraction were performed during and post-RT (99). Spontaneous ORN occurred 6 to 24 months after RT, whereas trauma-induced ORN had two frequency peaks: early, within 3 months of RT, related to trauma in a period of 14 days pre- and during RT; and late, 2 years post-RT (peak at 5 years) with a risk maintained over several years. On the other hand, Epstein et al. describe a more random phenomenon with a continuous risk at any time after RT, revealed by trauma (100). In a meta-analysis of post-RT extractions including 6,030 extractions in 37,805 patients (16%) in 21 studies (1986-2020), the incidence of ORN was 5.3% (95%CI, 2.9 to 8.2%) (89).

The ORN risk of dental extraction is minimized by performing extraction 21 days pre-RT and within 6 months post-RT, with fewer than five teeth extracted (2.4% compared with 12.1%, p<0.0001) (36).

In summary, ORN occurrence revealed by dental extraction after dentoalveolar infection is a continuous risk after RT. It is pivotal to avoid early ORN, the use of prolonged antibiotic prophylaxis (penicillin, clindamycin) around the post-RT dental extraction (31, 101), followed by a debated use of hyperbaric oxygen (31) or a 3-month pentoxifylline-tocopherol combination [ (102), personal communication].

### 6.3 Biopsy/surgery in irradiated tissue

Traumatic procedures such as tumor biopsy or surgery (13, 87) in an irradiated volume run the risk of additional factors associated with defective healing, which may be increased by previous extensive lymph node dissection linked to operative volume (bleeding, postoperative fibrosis) and lymphatic stasis.

In a recent retrospective study, 14.7% ORN occurred in 510 irradiated HNC patients: jaw resection significantly increased the likelihood of jaw ORN by up to 16% compared with RT alone, i.e., one in seven patients (103).

In a large retrospective study, complications after definitive surgery for jaw ORN were described in 49 patients (65%): minor complications (wound infection, wound dehiscence, skin graft failure, seroma, partial flap loss, and fistula) and major complications (washout for hematoma or infection, fistula, partial or total flap loss, and carotid blowout) (104).

## 7 Causal agent 4: infection-related factor—microbiota

How to think about the impact of infection parameters in ORN occurrence?

The oral cavity shelters a complex ecosystem with highly organized aggregates of microorganisms constituting the healthy oral microbiota, with many species (>750 bacteria and fungi) attached to the surfaces of teeth and oral mucosa in an extracellular matrix of biofilms (105). The distribution of these bacteria depends on oral ecological determinants as hard or soft substrate, and physicochemical (pH, redox potential, hormones…) and nutritional factors in a healthy balance: supragingival flora (*Streptococcus*, *Lactobacillus*, *Actinomyces*, *Pseudomonas*…) and subgingival flora, in periodontal pockets without oxygen, with Gram-negative anaerobes (*Fusobacterium*, *Bacteroides*, *Porphyromonas*, *Prevotella*, *Campylobacter*, *Tannerella*…) (106).

In oral cavity disease, rupture of this balance is characterized by the growth of certain microorganisms, the disappearance of some species, and the appearance of metabolites that start the infectious process: supragingival flora are responsible for demineralization of hard dental tissue, whereas subgingival flora are implicated in periodontal disease. However, high interindividual species richness and diversity are observed (107, 108).

In localized infection of irradiated mandible, as alveolitis or limited osteitis (**Figure 5**), diagnosis is often delayed, whereas the microbiota is remodeled in unbalanced disease favored by ischemic conditions (109–110). With the progression of ORN, including bone exposure and sequestra (**Figure 4**), the bacterial composition changes, with predominance of several concomitant bacteria (meaning four microorganisms (111)), some of them not cultivable, from predominant aerobes of stage I (*Streptococcus* 13%, *Prevotella* 13%, *Fusobacterium* 13%) to predominant Gram-negative anaerobes of stage III (112).

In chronic osteomyelitis, bacterial diversity in necrotic tissues does not significantly differ between patients treated with or without antibiotics, with a poor success rate when using only antimicrobial therapy (113–115). This infection is localized but persistent, in relation to a sequestrum in the necrotic area allowing intermittent exacerbation or purulent discharge (116–118). Prolonged administration of adapted antibiotics often fails to control this infection, despite their good surface effect, whereas microbial cultures have no specific identified pathogens (110).

A recent analysis of tissue culture from jaw ORN at the time of rescue flap surgery showed 77% cases (20/26 cases) with microbial growth: 58% bacterial species (multibacterial in 27%), 35% fungal, both 15% (119).

The architecture of heterogeneous and hypovascularized ORN tissues, and even more the presence of bone sequestra, hinder the penetration of medical drugs, thus mechanically protecting residual bacteria at depth against antibiotics (9, 120). In daily management of ORN, rather than true genetic antibiotic resistance, what is generally observed is that antibiotics are hindered mechanically from reaching bacteria protected deep within an inert bone matrix with branching caverns.

Chen et al. treated 322 patients with chronic suppurative osteomyelitis of the jaw and quantified a significant risk of recurrence of sepsis within a year and life-threatening complications: conservation of pathogenic teeth 32.4% (vs. 12.7% if extraction; p=0.001); infection at the ramus location 30.8% (vs. 11.5% at the anterior or posterior mandible; p=0.003); preexisting diabetes 29.3% (vs. 5.3% without); and inadequate previous antibiotic spectra and duration 20.3% (versus 8.5% adapted-prolonged treatment duration; p=0.001) (121).

In summary, when irradiated tissue is exposed, the oral microbiota becomes unbalanced, with altered composition of supra- (periapical) and subgingival (periodontal) flora, and development of a mean of four predominant bacteria. In the presence of bone sequestra, bacteria are mechanically more inaccessible to antibiotics (intermittent purulent discharge) rather than truly resistant: prolonged antibiotic treatment should control local infection better and allow “mechanical” access by further antifibrotic treatment.

## 8 Causal agent 5: vascular-related factors

What to think of the impact of macro- and microvascular parameters on ORN occurrence?

### 8.1 Patient-related factors

Tobacco and alcohol use is a traditional risk factor for HNC, particularly for oral cavity (OCC) and oropharyngeal (OPC) cancers. In the UK, smoking rates have declined from 46% (1974) to 20% (2010), whereas alcohol consumption has increased from 3.9 (1950) to 9.4 L/year (2004) (47). Patients who continue to smoke during RT have a 32% increased risk of ORN (122). Alcohol has less consistently been identified as a risk factor. However, 75% of patients with OCC and OPC who continue to drink alcohol during RT are 3.2 times more likely to develop ORN (39).

Vascular comorbidities. Vascular factors, as uncontrolled hypertension or unbalanced diabetes, may accelerate the onset of ORN.

### 8.2 Radiation-induced vascular factors

Vascular factors responsible for RI sequelae have very different causes and consequences: from capillaries inside the irradiated tissue (microvascular), to irradiated branches and stenosis of the carotid artery trunk (macrovascular).

a- An old concept of RI vascular fibrosis based on a theory of gradual ischemia–hypoxia was developed to account for capillary network destruction after RT. More recent ideas consider reactions of endothelial cells to RT, with procoagulant proinflammatory effects of thrombin and changed micro-vascularization, in relation to intermittent rather than chronic hypoxia inducing hypoxia-inducible factors, and then neo-angiogenesis possibly leading to telangiectasia (3). However, although these vascular dysfunctions are involved in generating RI fibrosis, their role seems indirect in the established fibrotic phase. The fibroblastic stromal concept sheds further light on RI fibrosis, in terms of continuous attack by reactive oxygen species leading to fibrogenesis.

In the 1980s, a 3H theory of ORN was developed (5): “hypoxic–hypocellular–hypovascular” tissue, considering that microorganisms only play a contaminant role. Partial oxygen pressure (pO_2_) was measured in irradiated cervical skin versus non-irradiated thoracic contralateral skin in 112 patients, reflecting a linear relationship between capillary index density and the risk of mandibular ORN. However, an experimental study in rabbits showing an immediate decrease in subcutaneous pO_2_ in the acute edematous leg after RT did not show any delayed skin modification in the fibrotic phase (123). This was confirmed by normal pO_2_ skin measures in 100 irradiated patients (124). Whereas subcutaneous tissue is very sensitive to oxygen, bone marrow reacts partially and slowly to hyperbaric oxygen, whereas ischemia makes bone more vulnerable to infections (125). In fact, these studies measured oxygen parameters, but not the true vascular defect.

A 2016 study using dynamic contrast-enhanced MRI parameters in 30 patients showed a significantly higher degree (×3) of leakiness in the mandibular vasculature of areas of advanced ORN, versus healthy mandible (38).

b- In a 1990 study, histopathological descriptions of RI necrosis suggested a hypovascular role in damaged bone tissue. Ischemic pathogenesis of mandibular ORN was reported in 17 patients versus 4 non-RT and 2 RT patients without ORN (126). While irradiated bone presented signs of periosteum fibrosis, cortical bone showed necrosis with sequestra, and bone marrow was replaced by arterial fibrosis and thrombosis. It was hypothesized that there is a weak premolar–molar–retromolar anatomical region, mainly vascularized by the inferior alveolar artery (branch of maxillary artery from external carotid artery) with stenosis, which is not compensated by the facial artery branches, which are also irradiated (**Figure 4**). Moreover, we think that, for some patients, this ischemia is worsened mechanically by the irradiated bone inside the mandibular canal, ride of the inferior alveolar artery (from mandibular foramen running obliquely downward in the ramus, and then horizontally forward in the body to the mental foramen) (**Figure 5**).

More recently, studies of the epidemiology of vascular diseases that measured the risk of cerebrovascular accidents by focusing on RI carotid arteriopathy have helped assess RI ischemic consequences. In a review of the literature on RI carotid artery stenosis (125), seven of nine studies (2002-2009) reported an increased incidence of stroke with a 5.6 relative risk after HNC. We think that for lesions of the external carotid artery, measurement tools are less easy to assess (facial versus cranial vascularization), while subjecting the carotid artery to the same cervical RT (127, 128). The risk of asymptomatic RI carotid artery stenosis documented by Doppler showed a prevalence of 16% to 55%, mostly at the carotid bifurcation (125). In asymptomatic HNC survivors after unilateral neck RT, there is an increased risk of carotid artery stenosis (8 RI versus 3 non-irradiated/44), which is enhanced by combined lymph node dissection, especially after 10 years (129, 130). Finally, a meta-analysis shows that Doppler assessment of carotid intima-media thickness, which is an intermediate phenotype for early atherosclerosis, is a strong predictor of future vascular events, with a relative risk of stroke 1.32 (131). After hemi-cervical RT for HNC, a 35% increase in intima-media thickness (contralateral control) was measured at 100 months: 0.30-mm increase with RT versus non-RT (132).

In summary, vascular factors are implicated in ORN occurrence—worsening via ischemia: microvascular fibrotic effect (capillaries) and macrovascular effect (arteriopathy of carotid branches).

## 9 Conclusion- early mouth-ORN management

This review does not focus on the primary prevention of ORN, as its incidence today is very low thanks to technical advances in radiotherapy. Instead, we have tried to identify the conditions for onset of early ORN and its destabilizing factors with a view to secondary prevention.

ORN is a complex disease that combines five main interwoven causal agent families, the weighting of each agent varying in a given patient over time and from one patient to another, agents worsening each other over time: radiation-induced fibrotic atrophy of the bone, periodontal disease and dental trauma, infection, and vascular factors.

Medical care focusing on comorbidity factors such as oral hygiene, infection control by short antibiotic use, and devitalized tissue removal are the usual necessary but often insufficient approaches to symptomatic management, potentially combined with limited dental extraction.

We consider that treatment of “early ORN” with curative intent should combine concomitant and synergistic control of these causal parameters: dental care, infection, fibrosis–necrosis, and vascular aspects, based on pathophysiological understanding of radiation-induced fibro-atrophy, improved bone healing, and arrest of progression to severe ORN.

## References

[1] Miller K Nogueira L Devasia T Mariotto A Yabroff K Jemal A . Cancer treatment and survivorship statistics, 2022. CA Cancer J Clin. (2022) 72:409–436. doi: 10.3322/caac.21731 35736631

[2] Regaud C . Sur la sensibilité du tissu osseux normal vis à vis des rayons X et gamma et sur le mécanisme de l’ostéoradionécrose. C R Soc Biol. (1922) 87:629–632.

[3] Delanian S Lefaix J-L . The radiation-induced fibro-atrophic process: therapeutic perspective via the antioxidant pathway. Radiother Oncol. (2004) 73:119–131. doi: 10.1016/j.radonc.2004.08.021 15542158

[4] Delanian S Lefaix J-L . Current management for late normal tissue injury: radiation-induced fibrosis and necrosis. Sem Radiat Oncol. (2007) 17:99–107. doi: 10.1016/j.semradonc.2006.11.006 17395040

[5] Marx R . A new concept in treatment of osteoradionecrosis. J Oral Maxillofac Surg. (1983) 41:351–357. doi: 10.1016/s0278-2391(83)80005-6 6574217

[6] Epstein J Wong F Stevenson-Moore P . Osteoradionecrosis: clinical experience and a proposal for classification. J Oral Maxillofac Surg. (1987) 45:104–110. doi: 10.1016/0278-2391(87)90399-5 3468211

[7] Støre G Boysen M . Mandibular osteoradionecrosis: clinical behaviour and diagnostic aspects. Otolaryngol Allied Sci. (2000) 25:378–384. doi: 10.1046/j.1365-2273.2000.00367.x 11012651

[8] Notani K Yamazaki Y Kitada H Sakakibara N Fukada H Omori K . Management of mandibular osteoradionecrosis corresponding to the severity of osteoradionecrosis and the method of radiotherapy. Head Neck. (2003) 25:181–186. doi: 10.1002/hed.10171 12599284

[9] Delanian S Chatel C Porcher R Depondt J Lefaix J-L . Complete restoration of refractory mandibular osteoradionecrosis by prolonged treatment with a pentoxifylline-tocopherol-clodronate combination (PENTOCLO): phase II trial. Int J Radiat Oncol Biol Phys. (2011) 80:832–839. doi: 10.1016/j.ijrobp.2010.03.029 20638190

[10] Tsai CJ Hofstede TM Sturgis EM Garden A Lindberg M Wei Q . Osteoradionecrosis and radiation dose to the mandible in patients with oropharyngeal cancer. Int J Radiat Oncol Biol Phys. (2013) 85:415–420. 10.1016/j.ijrobp.2012.05.032 22795804

[11] Lyons A Osher J Warner E Kumar R Brennan P . Osteoradionecrosis—a review of current concepts in defining the extent of the disease and a new classification proposal. Br J Oral Maxillofac Surg. (2014) 52:392–395. doi: 10.1016/j.bjoms.2014.02.017 24725905

[12] Watson EE Hueniken K Lee J Huang SH El Maghrabi A Xu W . Development and standardization of an osteoradionecrosis classification system in head and neck cancer: implementation of a risk-based model. J Clin Oncol. (2024) 42:1922–1933. doi: 10.1200/jco.23.01951 38691822 PMC11500043

[13] Peterson D Koyfman S Yarom N Duch Lynggaard C Ismaila N Forner L . Prevention and management of osteoradionecrosis in patients with head and neck cancer treated with radiation therapy: ISOO-MASCC-ASCO guideline. J Clin Oncol. (2024) 42:1975–1996. 38691821 10.1200/JCO.23.02750

[14] Miyamoto I Tanaka R Kogi S . Clinical diagnostic imaging study of osteoradionecrosis of the jaw: a retrospective study. J Clin Med. (2021) 10:4704. doi: 10.3390/jcm10204704 34682827 PMC8538245

[15] Mallya S Tetradis S . Imaging of radiation and medication-related osteonecrosis. Radiol Clin North Am. (2018) 56:77–89. doi: 10.1016/j.rcl.2017.08.006 29157550 PMC5726810

[16] Akashi M Wanifuchi S Kusumoto J Kishimoto M Kakei Y Hashikawa K . Potential role of post-treatment follow-up FDG-PET CT to detect mandibular osteoradionecrosis: a case report. Mol Clin Oncol. (2018) 8:61–67. doi: 10.3892/mco.2017.1477 29387398 PMC5769210

[17] Hakim S Bruecker C Jacobsen H Hermes D Lauer I Eckerle S . The value of FDG-PET and bone scintigraphy with SPECT in the primary diagnosis and follow-up of patients with chronic osteomyelitis of the mandible. Int J Oral Maxillofac Surg. (2006) 35:809–816. doi: 10.1016/j.ijom.2006.03.029 16829036

[18] Dinnoo A Benmoussa N Bidault F Brau JJ Tissot H Temam S . 18F-FDG PET/CT for early response assessment of jaw osteoradionecrosis after the PENTOCLO protocol: a promising imaging modality. J Stomatol Oral Maxillofac Surg. (2022) 123:e192–e198. doi: 10.1016/j.jormas.2021.11.005 34826634

[19] Moreno AC Watson EE Humbert-Vidan L Peterson D van Dijk L Guerrero-Urbano T . International expert-based consensus definition, classification criteria, and minimum data elements for osteoradionecrosis of the jaw: an interdisciplinary modified Delphi study. Int J Radiat Oncol Biol Phys. (2025) 122:341–354. doi: 10.1016/j.ijrobp.2024.12.017 39826846 PMC12303726

[20] Chronopoulos A Zarra T Troltzsch M Mahaini S Ehrenfeld M Otto S . Osteoradionecrosis of the mandible: a ten year single-center retrospective study. J Craniomaxillofac Surg. (2015) 43:837–846. doi: 10.1016/j.jcms.2015.03.024 25939311

[21] Grant B Fletcher G . Analysis of complications following megavoltage therapy for squamous cell carcinomas of the tonsillar area. Am J Roentgenol Radium Ther Nucl Med. (1966) 96:28–36. doi: 10.2214/ajr.96.1.28 4954881

[22] Murray C Herson J Daly T Zimmermans S . Radiation necrosis of the mandible: a 10 years study. Part I: factors influencing the onset of necrosis. Int J Radiat Oncol Biol Phys. (1980) 6:543–548. 10.1016/0360-3016(80)90380-6 7410128

[23] Morrish RB Chan E Silverman S Jr Meyer J Fu KK Greenspan D . Osteonecrosis in patients irradiated for head and neck carcinoma. Cancer. (1981) 47:1980–1983. 10.1002/1097-0142(19810415)47:8<1980::aid-cncr2820470813>3.0.co;2-y 7226091

[24] Withers H Peters L Taylor J Owen J Morisson W Schultheiss T . Late normal tissue sequelae from radiation therapy for carcinoma of the tonsil: patterns of fractionation study of radiobiology. Int J Radiat Oncol Biol Phys. (1995) 33:563–568. doi: 10.1016/0360-3016(95)00229-r 7558944

[25] Reuther T Schuster T Mende A Kubler A . Osteoradionecrosis of the jaws as a side effect of radiotherapy of head and neck tumour patients—a report of a thirty-year retrospective review. Int J Oral Maxillofac Surg. (2003) 32:289–295. doi: 10.1054/ijom.2002.0332 12767877

[26] Chang D Sandow P Morris C Hollander R Scarborough L Amdur R . Do pre-irradiation dental extractions reduce the risk of osteoradionecrosis of the mandible? Head Neck. (2007) 29:528–536. doi: 10.1002/hed.20538 17230555

[27] Lee IJ Koom WS Lee CG Kim YB Yoo SW Keum KC . Risk factors and dose-effect relationship for mandibular osteoradionecrosis in oral and oropharyngeal cancer patients. Int J Radiat Oncol Biol Phys. (2009) 75:1084–1091. doi: 10.1016/j.ijrobp.2008.12.052 19327914

[28] Huang K Xia P Chuang C Weinberg V Glastonbury C Eisele D . Intensity-modulated chemoradiation for treatment of stage III and IV oropharyngeal carcinoma. The University of California–San Francisco experience. Cancer. (2008) 113:497–507. doi: 10.1002/cncr.23578 18521908

[29] Gomez D Estilo C Wolden S Zelefsky M Krauss D Wong R . Correlation of osteoradionecrosis and dental events with dosimetric parameters in intensity-modulated radiation therapy for head-and-neck cancer. Int J Radiat Oncol Biol Phys. (2011) 81:e207–e213. doi: 10.1016/j.ijrobp.2011.02.003 21570202

[30] Gevorgyan A Wong K Poon I Blanas N Enepekides D Higgins K . Osteoradionecrosis of the mandible: a case series at a single institution. J Otolaryngol Head Neck Surg. (2013) 42:46. doi: 10.1186/1916-0216-42-46 24025531 PMC3847525

[31] Nabil S Samman N . Incidence and prevention of osteoradionecrosis after dental extraction in irradiated patients: a systematic review. Int J Oral Maxillofac Surg. (2011) 40:229–243. doi: 10.1016/j.ijom.2010.10.005 21115324

[32] De Felice F Thomas C Patel V Connor S Michaelidou A Sproat C . Osteoradionecrosis following treatment for head and neck cancer and the effect of radiotherapy dosimetry: the Guy’s and St Thomas’ Head and Neck Cancer Unit experience. Oral Surg Oral Med Oral Pathol Oral Radiol. (2016) 122:28–34. doi: 10.1016/j.oooo.2016.01.007 27039003

[33] Kuhnt T Stang A Wienke A Vordermark D Schweyen R Hey J . Potential risk factors for jaw osteoradionecrosis after radiotherapy for head and neck cancer. Radiat Oncol. (2016) 11:101. doi: 10.1186/s13014-016-0679-6 27473433 PMC4967325

[34] Raguse J Hossamo J Tinhofer I Hoffmeister B Budach V Jamil B . Patient and treatment-related risk factors for osteoradionecrosis of the jaw in patients with head and neck cancer. Oral Surg Oral Med Oral Pathol Oral Radiol. (2016) 121:215–221. doi: 10.1016/j.oooo.2015.10.006 26703417

[35] De Maesschalck T Dulguerov N Caparrotti F Scolozzi P Picardi C Mach N . Comparison of the incidence of osteoradionecrosis with conventional radiotherapy and intensity-modulated radiotherapy: osteoradionecrosis and IMRT. Head Neck. (2016) 38:1695–1702. doi: 10.1002/hed.24505 27240700

[36] Kuo TJ Leung CM Chang HS Wu CN Chen WL Chen GJ . Jaw osteoradionecrosis and dental extraction after head and neck radiotherapy: a nationwide population-based retrospective study in Taiwan. Oral Oncol. (2016) 56:71–77. doi: 10.1016/j.oraloncology.2016.03.005 27086489

[37] Studer G Bredell M Studer S Huber G Glanzmann C . Risk profile for osteoradionecrosis of the mandible in the IMRT era. Strahlenther Onkol. (2016) 192:32–39. doi: 10.1007/s00066-015-0875-6 26265308 PMC4705130

[38] Chen J-A Wang C-C Wong Y-K Wang CP Jiang RS Lin JC . Osteoradionecrosis of mandible bone in patients with oral cancer-associated factors and treatment outcomes: osteoradionecrosis and oral cancer. Head Neck. (2016) 38:762–768. doi: 10.1002/hed.23949 25521838

[39] Owosho AA Tsai CJ Lee RS Freymiller H Kadempour A Varthis S . The prevalence and risk factors associated with osteoradionecrosis of the jaw in oral and oropharyngeal cancer patients treated with IMRT: the Memorial Sloan Kettering Cancer Center experience. Oral Oncol. (2017) 64:44–51. doi: 10.1016/j.oraloncology.2016.11.015 28024723 PMC5560021

[40] Caparrotti F Huang S-H Lu L Bratman S Ringash J Bayley A . Osteoradionecrosis of the mandible in patients with oropharyngeal carcinoma treated with intensity-modulated radiotherapy. Cancer. (2017) 123:3691–3700. doi: 10.1002/cncr.30803 28608925

[41] Moon D Moon S Wang K Weissler M Hackman T Zanation A . Incidence of, and risk factors for, mandibular osteoradionecrosis in patients with oral cavity and oropharynx cancers. Oral Oncol. (2017) 72:98–103. doi: 10.1016/j.oraloncology.2017.07.014 28797468

[42] Mohamed A Hobbs B Hutcheson K Murri M Garg N Song J . Dose-volume correlates of mandibular osteoradionecrosis in oropharynx cancer patients receiving IMRT: results from a case-matched comparison. Radiother Oncol. (2017) 124:232–239. doi: 10.1016/j.radonc.2017.06.026 28733053 PMC5572506

[43] Aarup-Kristensen S Hansen C Forner L Brink C Eriksen JG Johansen J . Osteoradionecrosis of the mandible after radiotherapy for head and neck cancer: risk factors and dose-volume correlations. Acta Oncol. (2019) 58:1373–1377. doi: 10.1080/0284186x.2019.1643037 31364903

[44] Zhang W Zhang X Yang P Blanchard P Garden A Gunn B . Intensity-modulated proton therapy and osteoradionecrosis in oropharyngeal cancer. Radiother Oncol. (2017) 123:401–405. doi: 10.1016/j.radonc.2017.05.006 28549794 PMC5779856

[45] Yang Y Muller O Shiraishi S Harper M Amundson A Wong WW . Empirical relative biological effectiveness for mandible osteoradionecrosis in head and neck cancer patients treated with pencil-beam-scanning proton therapy: a retrospective, case-matched cohort study. Front Oncol. (2022) 12:843175. doi: 10.3389/fonc.2022.843175 35311159 PMC8928456

[46] Singh A Kitpanit S Neal B Yorke E White C Yom SK . Osteoradionecrosis of the jaw following proton radiation therapy for patients with head and neck cancer. JAMA Otolaryngol Head Neck Surg. (2023) 149:151–159. doi: 10.1001/jamaoto.2022.4165 36547968 PMC9912132

[47] Habib S Sassoon I Thompson I Patel V . Risk factors associated with osteoradionecrosis. Oral Surg. (2021) 14:227–235. doi: 10.1111/ors.1259740046247

[48] Oh H Chambers M Martin J Lim H Park H . Osteoradionecrosis of the mandible: treatment outcomes and factors influencing the progress of osteoradionecrosis. J Oral Maxillofac Surg. (2009) 67:1378–1386. doi: 10.1016/j.joms.2009.02.008 19531406

[49] Mohamed AS Abobakr M Tehami S Van Dijk LV Lai SY Fuller CD . Natural history and clinical/dosimetric determinants of osteoradionecrosis in a large cohort of head and neck cancer following curative radiotherapy: debunking the myth of decreased rates of osteoradionecrosis in the modern radiotherapy era. Int J Radiat Oncol Biol Phys. (2023) 117(S123):1054.

[50] Watson WL Scarborough JE . Osteoradionecrosis in intraoral cancer. Am J Roentgenol. (1938) 40:524–534.

[51] Meyer I . Infectious diseases of the jaws. J Oral Surg Am Dent Assoc. (1970) 28:17–26. 5262227

[52] Monnier Y Broome M Betz M Bouferrache K Ozsahin M Jaques B . Mandibular osteoradionecrosis in squamous cell carcinoma of the oral cavity and oropharynx: incidence and risk factors. Otolaryngol Head Neck Surg. (2011) 144:726–732. doi: 10.1177/0194599810396290 21493361

[53] Duarte VM Liu YF Rafizadeh S Tajima T Nabili V Wang MB . Comparison of dental health of patients with head and neck cancer receiving IMRT vs conventional radiation. Otolaryngol Head Neck Surg. (2014) 150:81–86. doi: 10.1177/0194599813509586 24145147

[54] Rosenthal D Chambers M Fuller C Rebueno N Garcia J Kies M . Beam path toxicities to non-target structures during intensity-modulated radiation therapy for head and neck cancer. Int J Radiat Oncol Biol Phys. (2008) 72:747–755. doi: 10.1016/j.ijrobp.2008.01.012 18455324 PMC9627578

[55] Frankart A Frankart M Cervenka B Tang AL Krishnan D Takiar V . Osteoradionecrosis: exposing the evidence not the bone. Int J Radiat Oncol Biol Phys. (2021) 109:1206–1218. doi: 10.1016/j.ijrobp.2020.12.043 33412258

[56] Rubin P Casarett G . Mature cartilage and bone in clinical radiation pathology (vol. II). In: Clinical Radiation Pathology (Vol. II). Philadelphia: WB Saunders Company (1968). p. 557–608.

[57] Bedwinek J Shukovsky L Fletcher G Daley T . Osteonecrosis in patients treated with definitive radiotherapy for squamous cell carcinomas of the oral cavity and naso-and oropharynx. Radiology. (1976) 119:665–667. doi: 10.1148/119.3.665 935406

[58] Setton J Caria N Romanyshyn J Koutcher L Wolden S Zelefsky MJ . Intensity-modulated radiotherapy in the treatment of oropharyngeal cancer: an update of the Memorial Sloan-Kettering Cancer Center experience. Int J Radiat Oncol Biol Phys. (2012) 82:291–298. doi: 10.1016/j.ijrobp.2010.10.041 21167652

[59] Ben-David M Diamante M Radawski J Vineberg K Stroup C Murdoch-Kinch CA . Lack of osteoradionecrosis of the mandible after intensity-modulated radiotherapy for head and neck cancer: likely contributions of both dental care and improved dose distributions. Int J Radiat Oncol Biol Phys. (2007) 68:396–402. doi: 10.1016/j.ijrobp.2006.11.059 17321069 PMC2702207

[60] Glanzmann C Gratz K . Radionecrosis of the mandibula: a retrospective analysis of the incidence and risk factors. Radiother Oncol. (1995) 36:94–100. doi: 10.1016/0167-8140(95)01583-3 7501817

[61] Thorn J Hansen H Specht L Bastholt L . Osteoradionecrosis of the jaws: clinical characteristics and relation to the field of irradiation. J Oral Maxillofac Surg. (2000) 58:1088–1093. doi: 10.1053/joms.2000.9562 11021701

[62] Schoen P Raghoebar G Bouma J Reintsema H Vissink A Sterk W . Rehabilitation of oral function in head and neck cancer patients after radiotherapy with implant-retained dentures: effects of hyperbaric oxygen therapy. Oral Oncol. (2007) 43:379–388. doi: 10.1016/j.oraloncology.2006.04.009 16996783

[63] Bak S Qi X Kelly J Alexander S Chung Y Gyurdzhyan S . Dosimetric distribution to tooth-bearing areas in intensity-modulated radiation therapy for head and neck cancer: a pilot study. Oral Surg Oral Med Oral Pathol Oral Radiol. (2016) 121:43–48. doi: 10.1016/j.oooo.2015.10.007 26679359

[64] Garg H Grewal M Rawat S Suhag A Bihari Sood P Grewal S . Dental pulp status of posterior teeth in patients with oral and oropharyngeal cancer treated with concurrent chemoradiotherapy. J Endod. (2015) 41:1830–1833. doi: 10.1016/j.joen.2015.08.006 26433856

[65] Walker M Wichman B Cheng A Coster J Williams K . Impact of radiotherapy dose on dentition breakdown in head and neck cancer patients. Pract Radiat Oncol. (2011) 1:142–148. doi: 10.1016/j.prro.2011.03.003 21857887 PMC3156461

[66] de Barros da Cunha S Fonseca F Ramos P Haddad C Fregnani E Aranha AC . Effects of different radiation doses on the microhardness, superficial morphology, and mineral components of human enamel. Arch Oral Biol. (2017) 80:130–135. doi: 10.1016/j.archoralbio.2017.04.007 28414987

[67] van Dijk L Abusaif A Rigert J Naser MA Hutcheson K Lai S . Normal tissue complication probability (NTCP) prediction model for osteoradionecrosis of the mandible in patients with head and neck cancer after radiation therapy: large-scale observational cohort. Int J Radiat Oncol Biol Phys. (2021) 111:549–558. doi: 10.1016/j.ijrobp.2021.04.042 PMC8906058 33965514

[68] Fajardo LF . Pathology of radiation injury. Locomotive system (Chap. 13). In: Masson Monographics in Diagnostic Pathology (MMDP). Masson: Strenberg (1982). p. 176–184.

[69] King M Casarett G Weber D . A study of irradiated bone: part I histopathologic and physiologic changes. J Nucl Med. (1979) 20:1142–1149. 536774

[70] Hoebers F Fergusson P O’Sullivan B . Bone. In human radiation injury (Chap 43). In: Shrieve DC Loeffler JS , editors. Human Radiation Injury. Philadelphia: Lippincott Williams Wilkins (2011). p. 481–498.

[71] Dekker H Schulten E Lichters I Van Ruijven L Van Essen H Blom GJ . Osteocyte apoptosis, bone marrow adiposity and fibrosis in the irradiated human mandible. Adv Radiat Oncol. (2022) 7:100951. 10.1016/j.adro.2022.100951 PMC9156996 35662809

[72] Dambrain R . La pathogénie de l’ostéoradionécrose. Rev Stomatol Chir Maxillofac. (1993) 94:140–147. 8337586

[73] Gal T Munoz-Antonia T Muro-Cacho C Klotch D . Radiation effects on osteoblasts *in vitro*: a potential role in osteoradionecrosis. Arch Otolaryngol Head Neck Surg. (2000) 126:1124–1128. doi: 10.1001/archotol.126.9.1124 10979127

[74] Curi M Dib L . Osteoradionecrosis of the jaws: a retrospective study of the background factors and treatment in 104 cases. J Oral Maxillofac Surg. (1997) 55:540–544. doi: 10.1016/s0278-2391(97)90478-x 9191633

[75] Qaisi M Montague L . Bone margin analysis for osteonecrosis and osteomyelitis of the jaws. Oral Maxillofac Surg Clin N Am. (2017) 29:301–313. doi: 10.1016/j.coms.2017.03.007 28709531

[76] Kim SG Jang HS . Treatment of chronic osteomyelitis in Korea. Oral Surg Oral Med Oral Pathol Oral Radiol Endod. (2001) 92:394–398. doi: 10.1067/moe.2001.117810 11598573

[77] Koga D Salvajoli J Alves F . Dental extractions and radiotherapy in head and neck oncology: review of the literature. Oral Dis. (2008) 14:40–44. doi: 10.1111/j.1601-0825.2006.01351.x 18173447

[78] Niewald M Fleckenstein J Mang K Holtmann H Spitzer W Rube C . Dental status, dental rehabilitation procedures, demographic and oncological data as potential risk factors for infected osteoradionecrosis of the lower jaw after radiotherapy for oral neoplasms: a retrospective evaluation. Radiat Oncol. (2013) 8:227. doi: 10.1186/1748-717x-8-227 24088270 PMC3851528

[79] Lockhart P Clark J . Pretherapy dental status of patients with malignant conditions of the head and neck. Oral Surg Oral Med Oral Pathol. (1994) 77:236–241. doi: 10.1016/0030-4220(94)90291-7 8170653

[80] Critchlow S Morgan C Leung T . The oral health status of pre-treatment head and neck cancer patients. Br Dent J. (2014) 216:E1. doi: 10.1038/sj.bdj.2013.1246 24413141

[81] Kojima Y Yanamoto S Umeda M Kawashita Y Saito I Hasegawa T . Relationship between dental status and development of osteoradionecrosis of the jaw: a multicenter retrospective study. Oral Surg Oral Med Oral Pathol Oral Radiol. (2017) 124:139–145. doi: 10.1016/j.oooo.2017.04.012 28606831

[82] Moraes R Dias F Figueredo C Fischer R . Association between chronic periodontitis and oral oropharyngeal cancer. Braz Dent J. (2016) 27:261–266. doi: 10.1590/0103-6440201600754 27224557

[83] Kielbassa A Hinkelbein W Hellwig E Meyer-Luckel H . Radiation-related damage to dentition. Lancet Oncol. (2006) 7:326–335. doi: 10.1016/s1470-2045(06)70658-1 16574548

[84] Moore C McLister C Cardwell C O’Neil C Donnelly M McKenna G . Dental caries following radiotherapy for head and neck cancer: a systematic review. Oral Oncol. (2020) 100:104484. doi: 10.1016/j.oraloncology.2019.104484 31786391

[85] Patel V Patel D Browning T Patel S McGurk M Sassoon I . Presenting pre-radiotherapy dental status of head and neck cancer patients in the novel radiation era. Br Dent J. (2020) 228:435–440. doi: 10.1038/s41415-020-1327-y 32221447

[86] Schuurhuis J Stokman M Witjes M Reintsema H Langendijk J Vissink A . Patients with advanced periodontal disease before intensity-modulated radiation therapy are prone to develop bone healing problems: a 2-year prospective follow-up study. Support Care Cancer. (2018) 26:1133–1142. doi: 10.1007/s00520-017-3934-y 29090383 PMC5847027

[87] Chrcanovic B Reher P Sousa A Harris M . Osteoradionecrosis of the jaws—a current overview—part 2: dental management and therapeutic options for treatment. Oral Maxillofac Surg. (2010) 14:81–95. doi: 10.1007/s10006-010-0205-1 20145963

[88] Wang T Liu C Chao T . Risk factors for and the role of dental extractions in osteoradionecrosis of the jaws: a national-based cohort study. Head Neck. (2017) 39:1313–1321. doi: 10.1002/hed.24761 28370713

[89] Beaumont S Bhatia N McDowell L Fua T McCullough M Celentano A . Timing of dental extractions in patients undergoing radiotherapy and the incidence of osteoradionecrosis: a systematic review and meta-analysis. Br J Oral Maxillofac Surg. (2021) 59:511–523. doi: 10.1016/j.bjoms.2020.10.006 33685773

[90] Hancock P Epstein JB Sadler G . Oral and dental management related to radiation therapy for head and neck cancer (review). J Can Dent Assoc. (2003) 69:585–590. 14653934

[91] Normando A Pérez-de-Oliveira M Guerra E Lopes MA Rocha AC Brandao TB . To extract or not extract teeth prior to head and neck radiotherapy? A systematic review and meta-analysis. Support Care Cancer. (2022) 30:8745–8759. doi: 10.1007/s00520-022-07215-y 35713725

[92] Wahl M . Osteoradionecrosis prevention myths. Int J Radiat Oncol Biol Phys. (2006) 64:661–669. doi: 10.1016/j.ijrobp.2005.10.021 16458773

[93] Lajolo C Gioco G Rupe C Troiano G Cordaro M Lucchese A . Tooth extraction before radiotherapy is a risk factor for developing osteoradionecrosis of the jaws: a systematic review. Oral Dis. (2020) 27:1–11. doi: 10.1111/odi.13485 32531873

[94] Gaggenheimer J Hoffman R . The importance of screening edentulous patients for oral cancer. J Prosthet Dent. (1994) 72:141–143. doi: 10.1016/0022-3913(94)90071-x 7932259

[95] Beech N Porceddu S Batstone M . Radiotherapy-associated dental extractions and osteoradionecrosis. Head Neck. (2017) 39:128–132. doi: 10.1002/hed.24553 27473832

[96] Grønhøj C Jensen D Dehlendorff C Nørregaard C Andersen E Specht L . Impact of time to treatment initiation in patients with human papillomavirus-positive and -negative oropharyngeal squamous cell carcinoma. Clin Oncol. (2018) 30:375–381. doi: 10.1016/j.clon.2018.02.025 29526405

[97] Beumer J Harrison R Sanders B Kurrasch M . Postradiation dental extractions: a review of the literature and a report of 72 episodes. Head Neck Surg. (1983) 6:581–586. doi: 10.1002/hed.2890060107 6629794

[98] Beumer J Harrison R Sanders B Kurrasch M . Osteoradionecrosis: predisposing factors and outcomes of therapy. Head Neck Surg. (1984) 6:819–827. doi: 10.1002/hed.2890060404 6706623

[99] Marx R Johnson R . Studies in the radiobiology of osteoradionecrosis and their clinical significance. Oral Surg Oral Med Oral Pathol. (1987) 64:379–390. doi: 10.1016/0030-4220(87)90136-8 3477756

[100] Epstein J Van der meij E McKenzie M Wong F Lepawsky M Stevenson-Moore P . Post-radiation osteonecrosis of the mandibule. Oral Surg Oral Med Oral Pathol. (1997) 83:657–662. doi: 10.1016/s1079-2104(97)90314-0 9195618

[101] Kanatas A Rogers S Martin M . A survey of antibiotic prescribing by maxillofacial consultants for dental extractions following radiotherapy to the oral cavity. Br Dent J. (2002) 192:157–160. doi: 10.1038/sj.bdj.4801322a 11863153

[102] Patel V Gadiwalla Y Sassoon I Sproat C Kwok J McGurk M . Prophylactic use of pentoxifylline and tocopherol in patients who require dental extractions after radiotherapy for cancer of the head and neck. Br J Oral Maxillofac Surg. (2016) 54:547–550. doi: 10.1016/j.bjoms.2016.02.024 26975577

[103] Hirsch J Krause I Tangmanee C Vorwerk H Stuck B Pitak-Arnnop P . Surgical predictors of osteoradionecrosis in irradiated head and neck cancer: jaw resection increases risk whereas neck dissection does not. J Stomatol Oral Maxillofac Surg. (2025) 126:102538. doi: 10.1016/j.jormas.2025.102538 40854456

[104] Contrera K Chinn S Weber R Roberts D Myers J Lai S . Outcomes after definitive surgery for mandibular osteoradionecrosis. Head Neck. (2022) 44:1313–1323. doi: 10.1002/hed.27024 35238096 PMC9081223

[105] Smoot L Smoot J Smidt H . DNA microarrays as salivary diagnostic tools for characterizing the oral cavity's microbial community. Adv Dent Res. (2005) 18:6–11. doi: 10.1177/154407370501800103 15998937

[106] Hallmer F Bjørnland T Andersson G Becktor J Kristoffersen A Enersen M . Bacterial diversity in medication-related osteonecrosis of the jaw. Oral Surg Oral Med Oral Pathol Oral Radiol. (2017) 123:436–444. doi: 10.1016/j.oooo.2016.11.011 28159588

[107] Socransky S Haffajee A . The bacterial etiology of destructive periodontal disease: current concepts. J Periodontol. (1992) 63(Suppl):322–331. doi: 10.1902/jop.1992.63.4s.322 29539682

[108] Goda A Maruyama F Michi Y Nakagawa I Harada K . Analysis of the factors affecting the formation of the microbiome associated with chronic osteomyelitis of the jaw. Clin Microbiol Infect. (2014) 20:309–317. doi: 10.1111/1469-0691.12400 24112138

[109] Reis Ferreira M Pasto A Ng T Patel V Urbano T Sears C . The microbiota and radiotherapy for head and neck cancer: what should clinical oncologists know? Cancer Treat Rev. (2022) 109:102442. doi: 10.1016/j.ctrv.2022.102442 35932549

[110] Li Z Fu R Huang X Wen X Zhang L . Oral microbiota may affect osteoradionecrosis following radiotherapy for head and neck cancer. J Transl Med. (2023) 21:391. doi: 10.1186/s12967-023-04219-y 37328857 PMC10276415

[111] Gudmundsson T Torkov P Thygesen T . Diagnosis and treatment of osteomyelitis of the jaw—a systematic review (2002-2015) of the literature. J Dent Oral Disord. (2017) 3:1066–1077. doi: 10.26420/jdentoraldisord.2017.1066

[112] Hansen T Kunkel M Kirkpatrick C Weber A . Actinomyces in infected osteoradionecrosis—underestimated? Hum Pathol. (2006) 37:61–67. doi: 10.1016/j.humpath.2005.09.018 16360417

[113] Andre C Khonsari R Ernenwein D Goudot P Ruhin B . Osteomyelitis of the jaws: a retrospective series of 40 patients. J Stomatol Oral Maxillofac Surg. (2017) 118:261–264. doi: 10.1016/j.jormas.2017.04.007 28502763

[114] Hudson J . Osteomyelitis of the jaws: a 50-year perspective. J Oral Maxillofac Surg. (1993) 51:1294–1301. doi: 10.1016/s0278-2391(10)80131-4 8229407

[115] Baur D Ali Altay M Flores-Hidalgo A Ort Y Quereshy F . Chronic osteomyelitis of the mandible: diagnosis and management—an institution’s experience over 7 years. J Oral Maxillofac Surg. (2015) 73:655–665. doi: 10.1016/j.joms.2014.10.017 25577460

[116] Ertas U Tozoglu S Gursan N . Chronic osteomyelitis: 20 years after mandible fracture. Dent Traumatol. (2004) 20:106–108. doi: 10.1111/j.1600-4469.2004.00229.x 15025693

[117] Birt M Anderson D Toby E Kng J . Osteomyelitis: recent advances in pathophysiology and therapeutic strategies. J Orthop. (2017) 14:45–52. doi: 10.1016/j.jor.2016.10.004 27822001 PMC5090239

[118] Mainous E Boyne J Hart G . Elimination of sequestrum and healing of osteonecrosis of the mandible after hyperbaric oxygen therapy. J Oral Surg. (1973) 31:336–339. 4512192

[119] Arienpour K Liu S Ciolek P Prendes B Fritz M . Mandibular osteoradionecrosis: defining the microbial milieu and antimicrobial resistance at the time of rescue flap surgery. Laryngoscope. (2024) 134:166–169. doi: 10.1002/lary.30785 37401754

[120] Schmitt SK . Osteomyelitis. Infect Dis Clin N Am. (2017) 31:325–338. doi: 10.1016/j.idc.2017.01.010 28483044

[121] Chen L Li T Jing W Tang W Tian W Li C . Risk factors of recurrence and life-threatening complications for patients hospitalized with chronic suppurative osteomyelitis of the jaw. BMC Infect Dis. (2013) 13:313. doi: 10.1186/1471-2334-13-313 23844897 PMC3711889

[122] Zevallos J Mallen M Lam C Karam-Hage M Blalock J Wetter D . Complications of radiotherapy in laryngopharyngeal cancer: effects of a prospective smoking cessation program. Cancer. (2009) 115:4636–4644. doi: 10.1002/cncr.24499 19569250 PMC9278696

[123] Aitasalo K Aro H . Irradiation-induced hypoxia in bones and soft tissues: an experimental study. Plast Reconstr Surg. (1986) 77:256–265. doi: 10.1097/00006534-198602000-00015 3080764

[124] Rudolph R Tripuraneni P Koziol J McKean-Matthews M Frutos A . Normal transcutaneous oxygen pressure in skin after radiation therapy for cancer. Cancer. (1994) 74:3063–3070. doi: 10.1002/1097-0142(19941201)74:11<3063::aid-cncr2820741126>3.0.co;2-c 7880243

[125] Gujral DM Chahal N Senior R Harrington KJ Nutting CM . Radiation-induced carotid artery atherosclerosis. Radiother Oncol. (2014) 110:31–38. doi: 10.1016/j.radonc.2013.08.009 24044796

[126] Bras J De Jonge H Van Merkesteyn J . Osteoradionecrosis of the mandible: pathogenesis. Am J Otolaryngol. (1990) 11:244–250. doi: 10.1016/0196-0709(90)90084-9 2240412

[127] Delanian S . Is radiation-induced arteriopathy in long-term breast cancer survivors an underdiagnosed situation? Critical and pragmatic review of available literature. Radiother Oncol. (2021) 157:163–174. doi: 10.1016/j.radonc.2021.01.009 33515666

[128] Shichita T Ogata T Yasaka M Yasumori K Inoue T Ibayashi S . Angiographic characteristics of radiation-induced carotid arterial stenosis. Angiology. (2009) 60:276–282. doi: 10.1177/0003319709335905 19497924

[129] Brown P Foote R McLaughlin M Halyard M Ballman K Collie A . A historical prospective cohort study of carotid artery stenosis after radiotherapy for head and neck malignancies. Int J Radiat Oncol Biol Phys. (2005) 63:1361–1367. doi: 10.1016/j.ijrobp.2005.05.046 16169673

[130] Plummer C Henderson R O’Sullivan J Read S . Ischemic stroke and transient ischemic attack after head and neck radiotherapy: a review. Stroke. (2011) 42:2410–2418. doi: 10.1161/strokeaha.111.615203 21817150

[131] Lorentz M Markus H Bots M Rosvall M Sitzer M Ebrahim S . Prediction of clinical cardiovascular events with carotid intima-media thickness. A systematic review and meta-analysis. Circulation. (2007) 115:459–467. doi: 10.1161/CIRCULATIONAHA.106.628875 17242284

[132] Dorresteijn L Kappelle A Scholz N Munneke M Schilma J Balm A . Increased carotid wall thickening after radiotherapy on the neck. Eur J Cancer. (2005) 41:1026–1030. doi: 10.1016/j.ejca.2005.01.020 15862751

